# Harlequin Ichthyosis Nanobubble Hydrotherapy: A Breakthrough in Treatment

**DOI:** 10.7759/cureus.68581

**Published:** 2024-09-03

**Authors:** Paul Stark, Heather Radigan, Yassar Aziz

**Affiliations:** 1 Health, London School of Economics, New York, USA; 2 Dermatology, Onondaga-Cortland-Madison Board of Cooperative Educational Services (OCM BOCES), New York, USA; 3 Dermatology, Al Dhannah Hospital, Abu Dhabi, ARE

**Keywords:** hydrotherapy, treatment, harlequin ichthyosis, breakthrough, nanobubble hydrotherapy

## Abstract

This case report details the management of a 10-year-old female pediatric patient with Harlequin ichthyosis (HI), a challenging skin disorder characterized by the production of 40 times the normal skin without trifats, leading to recurrent splits and fissures. Conventional treatments were ineffective until the introduction of nanobubbles. Encouraged by successful cases, the family engaged in a 100-hour medical trial conducted three days a week at the dermatologist's office, witnessing positive outcomes after the initial 20-minute session. The nanobubble’s success inspired the creation of the White Water Company in 2016, developing the first portable nanobubbler. Nanobubble technology not only addressed excessive skin production but also played a crucial role in biofilm remediation, offering a solution to an additional challenge. The 100-hour medical trial demonstrated commitment to the patient's well-being. The nanobubble bath completed the scratch-itch cycle by shedding skin during the bath and moisturizing the skin from the inside out, providing a holistic solution to the challenges posed by HI.

## Introduction

Harlequin ichthyosis (HI) is a rare and severe skin disorder marked by extensive thickening of the skin throughout the entire body. The first recorded instance of this condition dates back to 1750, documented by Reverend Oliver Hart. Its overall occurrence is infrequent, with an estimated incidence of one in 300,000 births [1-2].

HI represents a scarce and severe variant within the extensive spectrum of over 38 different types of ichthyosis. Despite lamellar ichthyosis (LI) being the most prevalent, the heightened severity in this genetic skin disorder spectrum is attributed to homozygous mutations in ABCA12, specifically linked to type 2 LI. The patients often present with rigid, armor-like plates of skin separated by deep fissures. The consequences encompass substantial skin deformation and extensive scaling, presenting various challenges, including an elevated risk of infection, difficulties in maintaining normal body temperature, and complications related to movement and breathing [3].

HI demands immediate and intensive care from birth, typically in a neonatal intensive care unit (NICU). Newborns with HI require specialized attention, including continuous monitoring and respiratory support. High morbidity and mortality rates, particularly in the initial months due to respiratory failure or sepsis, emphasize the critical nature of the condition, necessitating intensive interventions such as direct intubation.

The existing therapeutic approaches for HI predominantly revolve around systemic retinoid therapy, particularly acitretin, aiming to ameliorate digital and thoracic constrictions for enhanced respiratory function and improved mobility. However, these interventions pose potential risks, particularly in pediatric cases. Timely initiation is imperative, but the administration of systemic retinoids necessitates thorough pre-treatment assessments, encompassing various laboratory evaluations. Typically dosed between 0.5 and 1 mg/kg per day, acitretin requires careful titration based on continuous physical examinations and vigilant monitoring of potential side effects. Discontinuation of therapy at six months of age is feasible; nevertheless, the associated risks, including impacts on cholesterol, triglyceride levels, and metabolic parameters, underline the need for ongoing and cautious monitoring. In instances where oral therapy proves intolerable, a viable alternative involves topical retinoids, particularly 0.1% tazarotene cream. Despite these therapeutic options, long-term care for children with HI presents persistent challenges, necessitating regular follow-up and incorporating physical and occupational therapy to optimize the range of motion [4-5].

At present, treatment strategies, while comprehensive, primarily focus on symptomatic relief without directly addressing the underlying pathology. The need for intensive care, coupled with the risk of severe complications and the multifaceted nature of the disorder, underscores the limitations of existing therapeutic modalities, highlighting the urgency for innovative steps.

The nanobubble hydrotherapy emerges as a potential innovative step in addressing HI, particularly with its application in delivering oxygen and ozone. This has the potential to alleviate some of the complications associated with the disorder, including the discomfort caused by deep fissures and dense scales, contributing to overall symptom management [6].

## Case presentation

**Figure 1:**
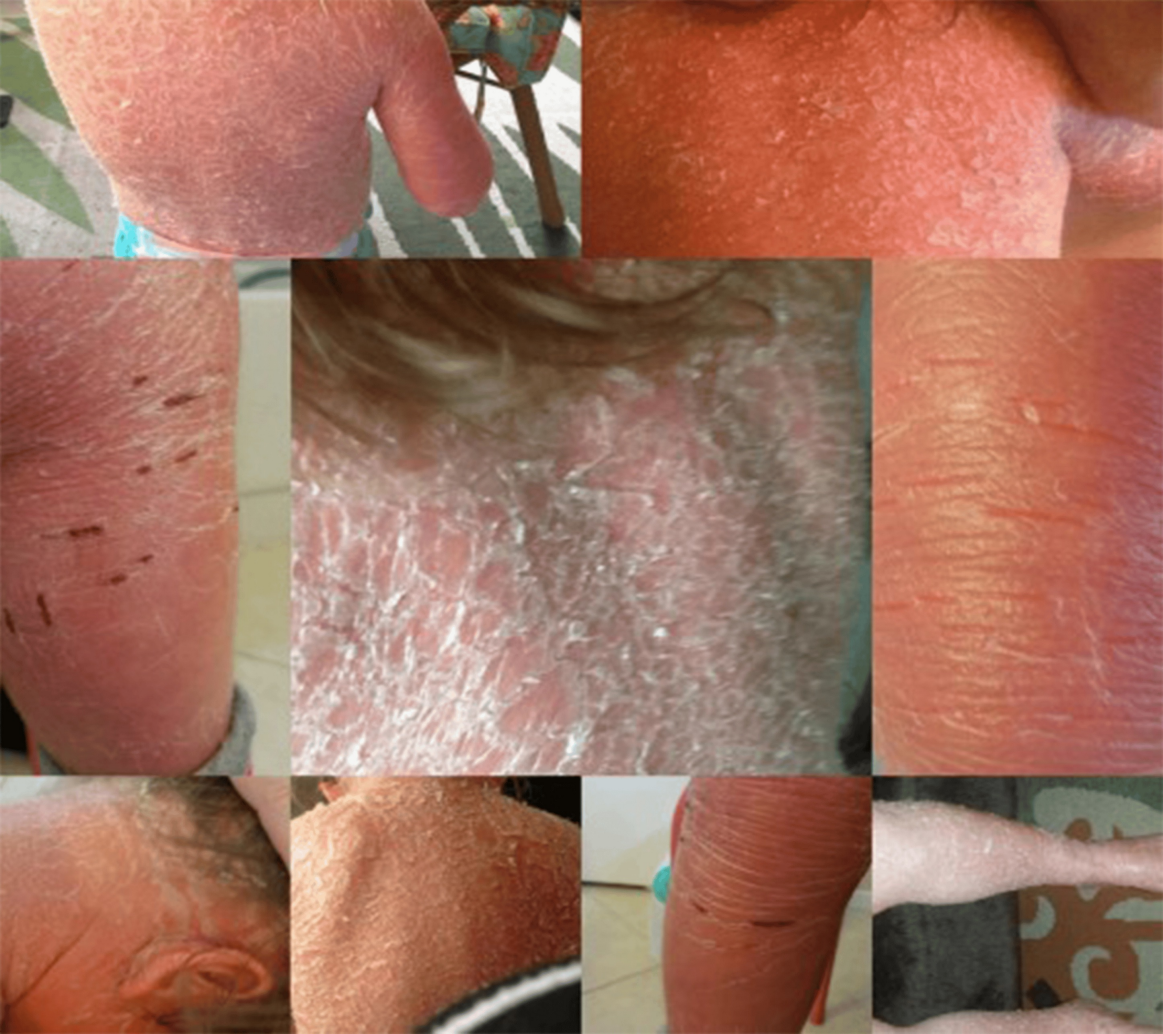
Before-therapy picture showing diffuse and scaly body rash

A 10-year-old female pediatric patient diagnosed with HI presented a unique challenge characterized by an accelerated rate of skin production. This heightened skin production, however, revealed inherent complications as the patient's skin lacked essential components crucial for its structural integrity, specifically trifats, elastin, and collagen. The patient's condition resulted in the production of 40 times the normal skin, leading to frequent cracks, splits, and fissures (Figure 1).

The intricate journey through the complexities of HI, with its lamellar presentation, involved the collaborative efforts of 27 medical professionals from various specialties. Five dermatologists played a pivotal role in recognizing the disorder's nuanced and multisystemic nature, particularly addressing the challenges related to skin shedding and debridement associated with the lamellar variant.

The symptomatic presentation extended beyond the remarkable skin hyperproliferation, encompassing challenges such as the inability to sweat, impaired temperature regulation, recurrent episodes of overheating, bilateral hearing loss, and complications associated with skin cracking, splitting, fissuring, and bleeding. These manifestations not only impacted the patient's physical well-being but also imposed a substantial emotional and psychological burden.

The complexity and severity of the symptoms underscored the imperative for a holistic and comprehensive approach to the patient's care, with a specific focus on mitigating the challenges posed by excessive skin shedding and debridement.

The treatment

For the initial 10 months of her life, attempts to address the patient’s condition proved challenging. Despite exhaustive efforts involving various bath additives, soaps, essential oils, and salts, no intervention completed the skin cycle, resulting in persistent complications. These complications included recurrent overheating, skin-impeding ear canals, and frequent infections due to skin cracking, splitting, fissuring, and bleeding. These complications align with common issues experienced by Ichthyosis patients.

The pivotal moment in the patient's treatment unfolded when her dermatologist introduced a groundbreaking microbubble sink. This cutting-edge technology, proven effective in treating conditions such as atopic dermatitis, psoriasis, and ichthyosis, marked a significant turning point. Recognizing the potential benefits, the patient's parents were motivated to delve into this innovative therapy. The treatment regimen involved an intensive 100-hour medical trial conducted three days a week at the dermatologist's office, showcasing its thorough evaluation and commitment to the patient's well-being. Notably, the trial demonstrated remarkable efficacy in eradicating pathogenic bacteria, including staph and methicillin-resistant *Staphylococcus aureus *(MRSA), underlining the broader antimicrobial benefits of the nanobubble hydrotherapy.

The outcome

**Figure 2:**
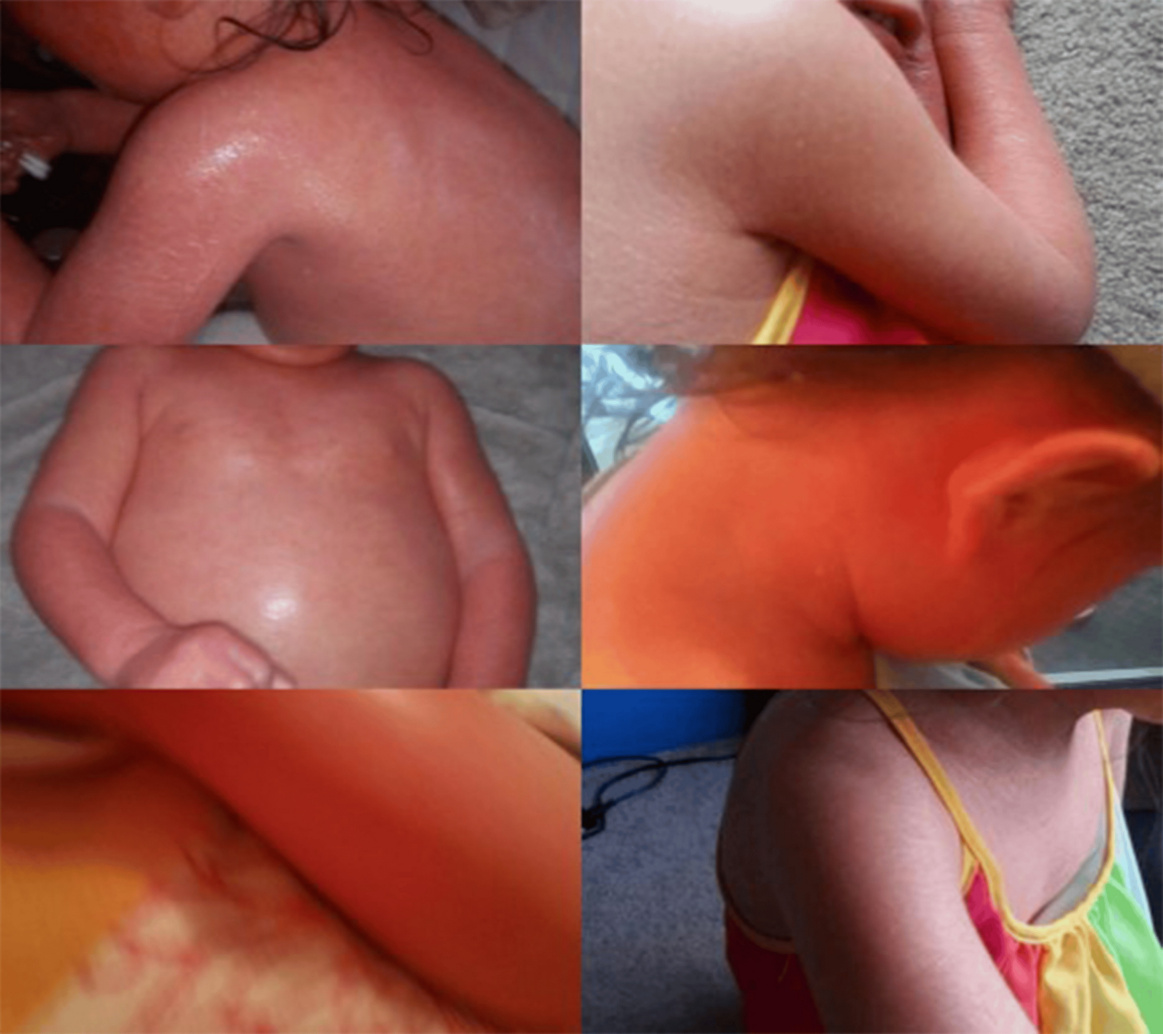
After-therapy picture showing resolution of the diffuse and scaly body rash

Remarkably positive outcomes became apparent, notably so after the initial 20-minute session. The excess skin of the pediatric patient underwent a shedding process, resulting in significantly smoother skin. Furthermore, all open wounds experienced effective cleansing and closure (Figure 2).

The transformative effects of the nanobubble hydrotherapy extended beyond the visible improvements, showcasing its impact on the pediatric patient's ears, skin, and immune system. The technology proved instrumental in remediating biofilm on the skin surface, eliminating the need for chlorine bleach baths. Notably, the portable solution also addressed mobility concerns.

The success story of the nanobubbles ignited a transformative journey for the pediatric patient's parents, who, fueled by the desire to expand the accessibility of this groundbreaking technology, embarked on a mission to innovate further. This journey led to the development of a portable solution and marked the inception of the White Water Company in 2016.

The nanobubble hydrotherapy not only facilitated biofilm remediation but also played a crucial role in open wounds' healing and oxygenation of blood. The nanobubbles, capable of delivering oxygen intravenously, contributed to the completion of the scratch-itch cycle by shedding skin in the bath and moisturizing the skin from the inside out. Unlike traditional methods relying on acids and retinoids, the nanobubble hydrotherapy proved to be a medication-free and effective alternative for patients with HI.

Origin of the White Water Company

Recognizing the need for collaboration with experts in the nanobubble field, the White Water Company engaged with industry leaders and sought guidance from specialists in the domain. In a strategic partnership with a world-leading industrial manufacturer, also a founding member of the Fine Bubble Association, the company harnessed collective expertise to create the world's first truly portable nanobubbles.

The White Water Company's journey, rooted in personal experience, has not only resulted in a transformative solution for the pediatric patient but has also contributed to advancing the field of nanobubble technology. By creating a portable Nanobubbler, the company aims to empower individuals within the ichthyosis community, providing them with a tool to manage their condition effectively.

The success of nanobubble therapy is attributed to the transformative capabilities of the portable Nanobubbler by the White Water Company. This revolutionary system saturates bathwater with billions of minuscule air bubbles, enriched with high levels of dissolved oxygen, and anions, and maintains consistent heat. Collectively, these elements create an additive-free bathing experience that not only enhances the skin but also induces relaxation throughout the body.

The properties of nanobubble bathing extend beyond a generally positive impact and specifically address the challenges faced by individuals dealing with skin diseases. The microscopic size and abundance of these bubbles allow for deep and thorough penetration into the skin, contributing to the shedding of excess skin and promoting overall skin health. The high levels of dissolved oxygen further aid in rejuvenating the skin, leaving it smoother and more vibrant.

## Discussion

The success story of the nanobubble hydrotherapy and the subsequent development of the White Water Company's portable Nanobubbler underscore the transformative potential of innovative therapies in managing complex dermatological conditions, particularly HI. The incorporation of the nanobubble hydrotherapy, particularly with its application in delivering oxygen and ozone, opens new avenues for treating HI.

Oxygen nanobubbles (OnBs) offer a novel approach to address hypoxia-related conditions, a critical challenge in HI. The thickened and cracked skin in HI can impede normal oxygenation, potentially exacerbating the condition. OnB's small size enables deeper penetration and more efficient oxygen delivery, offering a promising solution for the hypoxia associated with HI. This enhancement in oxygenation could potentially alleviate the metabolic crisis caused by skin barrier dysfunction, promoting tissue repair and reducing the severity of symptoms such as skin cracking and scaling [7].

HI patients, with their thickened and cracked skin, are highly susceptible to skin infections. Traditional treatment methods face challenges, especially with the rise of antibiotic-resistant bacteria. The studies by Hayakumo et al. and Gao-Feng Zha et al. highlight the potential of ozone nanobubbles to provide an effective solution to this problem. Ozone nanobubbles demonstrate strong antimicrobial properties, effectively combating a wide range of pathogens. Their ability to penetrate and deliver antimicrobial agents directly at the site of infection presents a significant advantage for HI patients, where conventional treatments may be hindered by the condition. This suggests a potential breakthrough in reducing the likelihood of infections and improving overall skin health in HI patients [8-9].

The nanobubble hydrotherapy, particularly in its capacity to enhance tissue oxygenation, holds promise for improving skin health in individuals with HI. The technology's impact on collagen synthesis, a crucial factor in skin repair and regeneration, is of particular significance. Chronic hypoxia, often experienced by HI patients due to their thickened skin, can impede collagen synthesis and reduce the expression of transforming growth factor-beta1 (TGF-β1), crucial for procollagen gene transcription. By enhancing skin oxygenation, nanobubbles could positively influence collagen synthesis, improving the texture and elasticity of the skin. This has the potential to reduce skin cracking and enhance skin barrier function through enhanced extracellular matrix (ECM) formation [6].

## Conclusions

This case study highlights crucial insights in managing HI and advancing innovative therapies. The transformative journey, from the success of the nanobubble hydrotherapy to the development of the White Water Company's portable Nanobubbler, underscores the influential role of personal experiences in driving innovation. Collaboration with leading experts in the nanobubble field highlights the significance of interdisciplinary collaboration and expertise in therapeutic development.

The nanobubble hydrotherapy, with its multifaceted potential including biofilm remediation, oxygen, and ozone nanobubbles, presents promising avenues for addressing key challenges associated with HI. From enhancing skin oxygenation to combating infections and promoting skin regeneration, nanobubble technology provides a comprehensive approach to managing the complex manifestations of HI. Tailoring a portable solution for the HI community reflects a commitment to improving individual outcomes and empowering patients and their families.
